# Physical activity on the mental health of children and adolescents during COVID-19 pandemic-induced school closures—A systematic review

**DOI:** 10.1371/journal.pone.0299158

**Published:** 2024-06-25

**Authors:** Bingbing Zhong, HaiChun Sun, Guixiang Wang, Shu Junwen, Shaohua Tang, Yuan Gao, Hanwen Chen, TianCi Lu, Jun Yan

**Affiliations:** 1 College of Physical Education, Yangzhou University, Yangzhou, China; 2 College of Education, University of South Florida, Tampa, FL, United States of America; 3 Department of Sports Work, Beijing University of Civil Engineering and Architecture, Beijing, China; University of Tartu, ESTONIA

## Abstract

**Propose:**

To review published Physical Activity (PA) on the Mental Health of Children and Adolescents aged 5 to 18 years during COVID-19 pandemic-induced school closures.

**Methods:**

From the database creation to April 2022, 10 databases are retrieved, with 4427 records filtered, 14 included in this research. The research takes Agency for Healthcare Research and Quality (AHRQ) evaluation standards.

**Results:**

The thesis selects 14 studies from 6 countries, involving 400009 children and adolescents. These studies happened during the lockdown of COVID-19 (from December 2019 to April 2021). During the lockdown of COVID-19, schools were closed, which was considered part of a more extensive lockdown. Schools were closed for 1 to 4 weeks. There were 10 high quality studies (71.4%) and 4 medium quality studies (28.6%). 4 studies report that the pandemic reduces the time of PA but increases the time of watching screen and sitting. 10 studies (71.4%) identify that PA is positive for the mental health, because it helps reduce mental symptoms to a certain extent, especially anxiety, depression, and emotional disorders. 5 studies show that PA may not improve the mental health of children and adolescents under 12 during the pandemic. 4 studies indicate that the influence of PA on mental health of children and adolescents is determined by the amount of activity, including the extent, intensity, frequency, and duration, etc.

**Conclusions:**

In this narrative synthesis of reports from the class suspension period, reports that PA has a improve on the mental health of children and adolescents to a certain extent. it is found that PA may be helpful in reducing mental health symptoms of children and adolescents who are influenced by class suspension because of the COVID-19 pandemic. Therefore, stakeholders of the mental health of children and adolescents around the world should recommend PA because it is a practicable and beneficial way for long-term mental support.

## Introduction

The COVID-19 pandemic is the biggest threat to public health in this century. Globally, as of 6:30 pm CEST, 16 August 2023, there have been 769,806,130 confirmed cases of COVID-19, including 6,955,497 deaths, reported to WHO (World Health Organization) [1]. In many countries and regions, lockdown and travel ban have been carried out to reduce the transmission of SARS-CoV-2 during the COVID-19 pandemic. Except for closing public places, such as school, restaurants, parks, shopping centers, etc., governments suggest people stay at home as much as possible. Theory and evidence suggest that closure of schools and restrictions not only influence on global medical health, economy, and society, but also between children and adolescents have the potential to be related to harms for students through a number of mechanisms, such as reduced access to school services and reduced contact with significant adults such as teachers, It directly breaks the regular daily life of children and adolescents, There may also be a range of associations owing to the loss of PA gained from active transport to school as well as from school sports [2–5].

During the lockdown, there was a notable rise in anxiety and depression among children and adolescents. This increase in mental health issues has been attributed to factors like social isolation, decreased physical activity, and heightened family stress. According to a review study [6], the COVID-19 pandemic and ensuing lockdowns have had a prolonged and varied impact on the psychosocial and mental well-being of young people, particularly affecting those with existing vulnerabilities. A separate study highlighted a global rise in depression and anxiety symptoms among children and adolescents during the COVID-19 pandemic [7]. While the prevalence and severity of these conditions varied across different studies, the overall trend showed a clear increase. A study in Germany examined the effect of COVID-19 on children and adolescents’ quality of life and mental health, revealing that the pandemic and lockdowns negatively impacted their mental well-being [8]. Moreover, the prevalence of anxiety, depression, stress, and other mental health issues has risen among adolescents in Low Middle Income Countries during the pandemic [9]. In China, the enforced home isolation during the pandemic heightened the risk of anxiety, depression, and PTSD among children and their families [10].

Similarly, It is worrying that around the world, children and adolescents are experiencing Physical Inactivity (PI) [11]. A recent study shows that a young person needs at least 60 minutes of Moderate-to-Vigorous Physical Activity (MVPA) [12] every day. However, in an investigation involving 105 countries and regions, 80.3% of adolescents aged 13 to 15 have less than 60 minutes of MVPA [13]. During the pandemic, most people sit for a longer time at their homes, especially adolescents, who have to study online for a long time, which leads to less Physical Activity (PA) and more time sitting in front of screens. Studies show that the PI of adolescents is related to the increase in anxiety, inefficiency, and risk of depression [14]. The COVID-19 pandemic and the following PI is an unprecedented health crisis and challenge for children and adolescents.

In the early stages of Omicron becoming the mainstream variant of the new coronavirus in late 2021, countries still chose cautious prevention and control strategies. However, after a significant reduction in the incidence of severe illness and case fatality in Omicron compared to the previous Delta variant was observed, most countries began to relax outbreak control measures from the end of February 2022. From February 24, 2022, the UK will lift all epidemic prevention and control measures in England, no longer quarantine COVID-19 infected people, and officially start to implement the "living with COVID-19" plan. In the United States, the CDC updated its epidemic prevention guidelines on February 25, 2022, determining that more than 60% of areas in the United States are at low risk of the epidemic, and the public does not need to wear masks in indoor places such as schools. In addition, France, Germany, Sweden and other countries have also lifted or significantly reduced COVID-19 control measures from March 2022. In March 2022, COVID-19 controls began to be lifted or relaxed in almost all countries [15].

It is worth noting that a previous review examining the impact of PA-based interventions on the mental health of children and adolescents in the context of the epidemic [16] highlighted the fact that nearly 60% of included studies (n = 7) reported statistically significant improvements at follow-up. However, most of the included studies (n = 16) in that review were quasi-experimental designs. Therefore, the causal relationship between PA and mental health in children and adolescents remains controversial. Since then, this field has received more and more research attention, so it is necessary to conduct a new and more thorough review.

We present here a narrative synthesis summarizing the available evidence from April 2022 in a systematic and all the articles from entire globe were included in the analysis manner to examine the impact of PA on the mental health of children and adolescents on school closures during COVID-19.

## Methods

We undertook a systematic review and narrative synthesis to answer the link between physical activity and Children and Adolescents mental health during COVID-19 pandemic-induced school closures.

We followed the relevant requirements of the Preferred Reporting Items for Systematic Reviews and Meta-analyses (PRISMA) reporting guideline [17], and our protocol was prospectively registered with PROSPERO (CRD42023449719).

### Search strategy

We searched 10 electronic databases (PubMed, PsycINFO, Scopus, EBSCO, Sport Discuss, CINAHL, Cochrane, Medline, Embase, Web of Science) from inception to October 2022, We used a combination of free-text controlled terms to identify citations containing children and adolescents concepts of either, physical activity, exercise, sports, COVID-19, epidemic, school closure, restricted access to education, health behavior, mental health (Information in the S1 File). We screened the reference list of included articles and asked experts in the field for additional studies.

Inclusion criteria included any children and adolescents aged 5 to 18 years and school closure (any duration) in response to COVID-19 pandemic-induced school closures, whether together with broader nonpharmaceutical interventions (i.e, lockdown) or alone; higher education and school absences, truancy, and holidays were excluded; any form of PA performed during this period. Controlled studies (open schools or regions without lockdowns), uncontrolled pre-post studies (change from before closure), and cross-sectional studies (comparison with reference data) were included. Observational, cohort, uncontrolled pre-post, modeling, and cross sectional published or preprint studies and reports with prespecified outcomes in English were included.

Four researchers (Zhong, B. B., Wang, G. X., Gao,Y., Tang, S.H.) shall filter the retrieved result separately according to the title or abstract, and then decide whether it should be included in the evaluation after accessing the whole paper. Next, the title is filtered by a reviewer (Yan, J.). In the end, whether the paper is included is decided by the Four authors.

### Data extraction and quality evaluation

The data are extracted by Four authors (Zhong, B. B., Wang, G. X., Chen, H. W., Lu, T. C.) and examined by another author (Sun, H. C.). In order to evaluate the quality of each research, the Four authors (Zhong, B. B., Wang, G. X., Chen, H. W., Lu, T. C.) shall evaluate each research separately to ensure its reliability. When the five of them have a disagreement, another evaluator (Sun, H. C.) shall solve the disagreement.

According to the evaluation criteria for the cross-sectional studies published by Agency for Healthcare Research and Quality (AHRQ), the quality score of each research ranges from 0 to 11. In this research, 4 papers are high-quality (scored 8–11), 10 papers are medium-quality (scored 4–7); and no paper is low-quality (scored 0–3). The evaluation criteria are related to the selection of research subjects, the subjective factors of the evaluator, the integrity of the data and the explanation of the deficiency, and the control of confounding factors.

### Data collation and analysis

Owing to heterogeneity of designs and measures, meta-analysis was not possible. Therefore, the result is presented in narrative comprehension. The following message is settled in an information in the S1 File with the help of Microsoft Excel: (1) the date and nationality of publication; (2) the details of methodology (such as the design of the research, the characteristics of the samples, the time of investigation, the duration and the follow-up period of intervention; and the way of investigation); (3) the key results of the influence of PA on mental health (such as mitigating anxiety and improving mental health).

## Results

**Fig 1:**
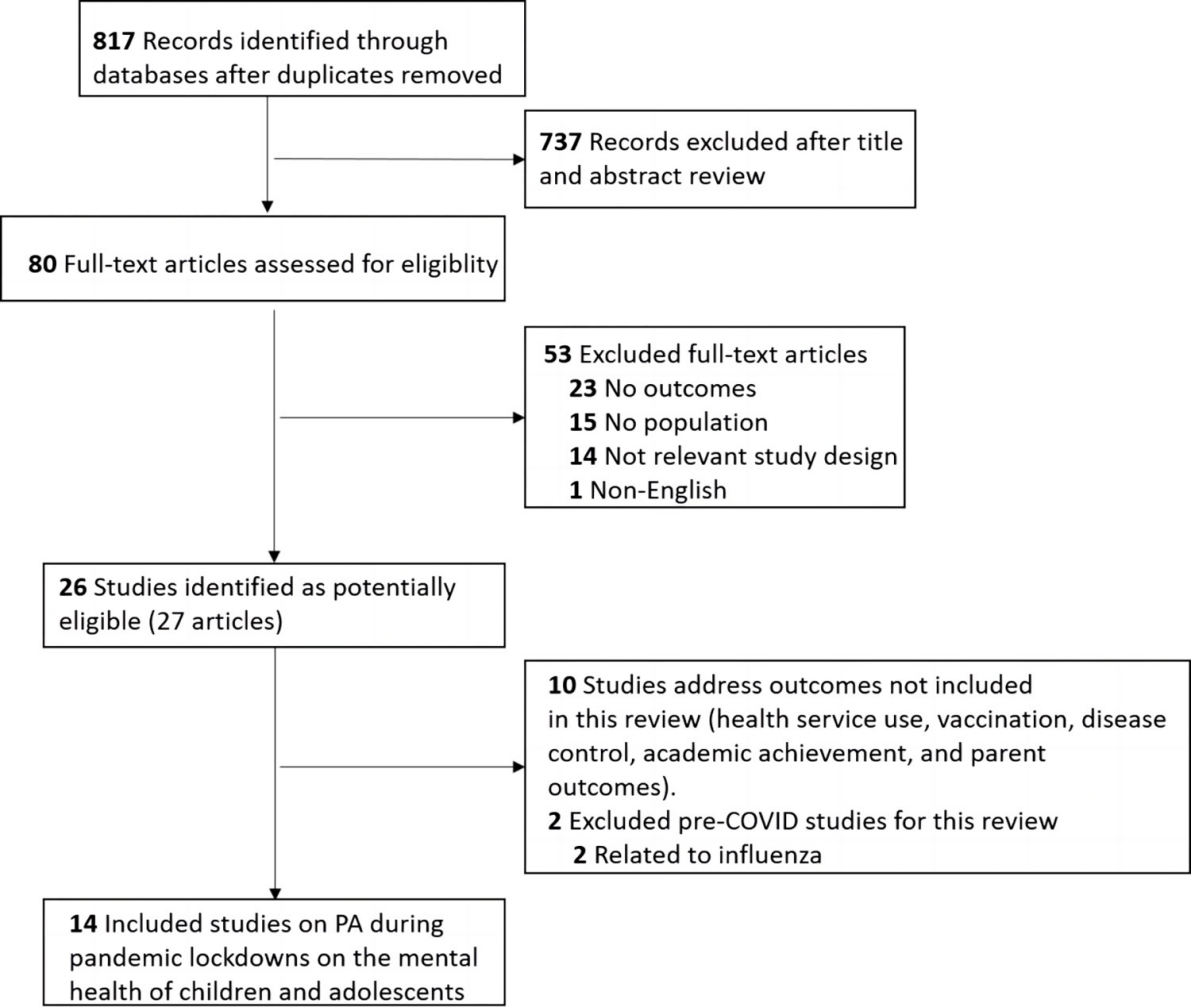
PRISMA flow diagram.

In 10 databases, a total of 4427 papers are retrieved and 817 papers are left after removing duplicates, of which 80 were reviewed in full text, and 27 studies (reported in 26 publications) were judged to be potentially relevant. Fig 1 shows the search flow. Here we report findings from 14 studies reporting PA during pandemic lockdowns on the mental health of children and adolescents.

The 14 papers of which 4 papers are high-quality, 10 papers are medium-quality, and no paper is low-quality. The result is shown in Table 1.

**Table 1:** Characteristics of included studies.

AHRQ quality analysis														
	Szwarcwald et al [24] 2021	Awais et al [25] 2021	Alves et al [28] 2021	Mcguine et al [18] 2021	Kang et al [26] 2021	Mcguine et al [16] 2022	Villodres et al [21] 2021	Xiao et al [19] 2021	Mcarthur et al [22] 2021	Tandon et al [29] 2021	Mitra et al [23] 2021	Ren et al [20] 2021	Maximove et al [27] 2022	Alve et al [30] 2020
1) Define the source of information(survey, record review)	1	1	1	1	1	1	1	1	1	1	1	1	1	1
2) List inclusion and exclusion criteria for exposed and unexposed subjects(cases and controls) or refer to previous publications	1	1	1	1	1	1	1	1	1	1	1	1	1	1
3) Indicate time period used for identifying patients	1	1	1	1	1	1	1	1	1	1	1	1	1	1
4) Inaicate wnetner or not supjects were consecutive if not population-based	0	0	1	0	0	0	0	1	0	0	0	0	0	0
5) Indicate if evaluators of subjective components of study were masked to other aspects of the status of the participants	0	1	1	1	0	1	-	1	-	1	0	0	0	0
6) Describe any assessments undertaken for quality assurance purposes (e.g., test/retest of primary outcome measurements)	0	1	1	1	1	1	1	1	1	1	1	1	1	1
7) Explain any patient exclusions from analysis	0	1	1	1	0	1	0	0	1	0	0	1	0	0
8) Describe how confounding was assessed and/or controlled.	1	0	1	0	0	1	0	1	1	1	0	0	0	0
9) If applicable, explain how missing data were handled in the analysis	0	1	0	0	0	0	0	0	1	0	0	1	0	0
10) Summarize patient response rates and compieteness of data collection	0	blb	1	0	1	0	1	0	1	1	0	1	0	0
11) Clarify what follow-up, if any, was expected and the percentage of patients for which incomplete data or follow-up was obtained	-	-	1	-	-	-	-	-	-	1	-	-	-	1
Scores	4	8	10	6	5	7	5	7	8	8	4	7	4	5
Quality	Medium	High	High	Medium	Medium	Medium	Medium	Medium	High	High	Medium	Medium	Medium	Medium

1 represents yes and 0 represents no (The fifth item, by contrast, is 1 for no and 0 for yes)

Characteristics of the included papers are shown in Table 2. 4 papers examined the correlation between PA and mental health in children under 12, 5 papers study the the correlation between PA and mental health of adolescents above 12, and 5 papers study the correlation between PA and mental health of both children and adolescents. The subjects of these studies are healthy children and adolescents, mostly selected from the public or from schools. These studies are conducted in different countries, of which 5 are in America [16, 18, 28–30], 3 are in China [19, 20, 26], 1 in Spain [21], 3 in Canada [22, 23, 27], 1 in Brazil [24], and 1 in Pakistan [25]. These studies are conducted through questionnaires and investigations online or on the phone. In addition, there is a huge difference in the number of samples and the form of PA. most papers usually compare factors like whether PA is involved, the intensity of the PA, the frequency in one week, and the duration. The 14 studies adopt different ways to evaluate PA and mental health, but usually, the result is observed by the adolescents themselves or by children’s parents and questionnaires.

**Table 2:** Descriptive characteristics of included studies.

Source	Study design	Sample	Period of reference	Context	Results	Conclusion
Szwarcwald et al [24] 2021, Brazil	Cross-sectional survey	N = 9470; Age_range_:12-17years; Female:50.2%	June 27-september 17,2020	All of the participants were recruited from all states of Brazil; During COVID-19 pandemic, school closures and confinement; Consent was obtained from the participants or their guardians for the questionnaires, all of the responses were anonymous.	The prevalence of two or more self-reported emotional problems(frequent sadness, frequent irritability and sleep problems) was significantly higher among females (50.1%) than males (24.8%); Self-reported emotional problems were correlated with age significantly, the prevalence rate was 34.3 percent(12-15years old) and 44.5%(16-17years old); Physical activity for 60 min or over at least twice a week was inversely correlated with the emotion problem (OR = 0.82, *p*<0.001).	During COVID-19 pandemic, the intake of practice of physical activity showed a positive influence on emotional well-being; Growing screen time can have an impact on emotional well-being by increasing sedentary time; Emotional issues(frequent sadness, frequent irritability and sleep problems) were significantly more common among females.
Awais et al [25] 2021, Pakistan	Cross-sectional survey	N = 225; Age_range_:15-19years, M_age_ = 17.9years,SD = 1.22; Female:n = 118(52.4%)	March-May,2020	All of the participants were recruited from Army Public School and College(set in Sibi, Balochistan) during lockdown; Most participants (87.1%) had no relatives or acquaintances that were infected with COVID‑19.	College‑going adolescents with a decreased level of physical activity; The majority of the participants, 145 (64.4%), were likely to be suffering from psychological distress; There was a significant effect of physical activity (*p* < 0.001)on the levels of distress; A moderate negative correlation of physical activity (*r* = -0.340, *p*<0.001) was found with psychological distress levels.	Those students who were sleeping late at night, spending more time in front of screens, living a sedentary life, and had less sleep duration were more likely to be suffering from psychological distres; Students partaking in physical activity were found to be in lesser psychological distress; Students who slept late,woke up late and have higher screen time having a higher level of distress.
Alves et al [28] 2021, United States	Cross-sectional survey	N = 64; Age_range_:9-15years; Female:63%; Overweight/obesity (n = 30,M_age_ = 11.7years, SD = 1.2,Female:57%), Healthy weight (n = 34,M_age_ = 11.9years, SD = 1.2,Female:68%)	April 22-July 29,2020 (Phase 1:April 22-May 7, Phase 2:May 8-July 29)	All of the children were residents of California,which was under a statewide‘stay-at-home’ locking down staring 19 March 2020 (Phase 1:with mandatory order of‘stay-at-home’,Phase 2:public areas were allowed to reopen with restrictions,but reclosed on 13 July).	The anxiety scores of child:5standard deviations greater than normative values from paediatric populations prior to the pandemic; The reduce of anxiety level associated with higher positive affect and PA in overweight/obesity children,associated with higher positive affect in children with healthy Weight; Greater leisure screen time was associated with higher negative affect irrespective of child BMI status.	These associations highlight the potential mental health benefits of maintaining positive affect,engaging in PA and limiting leisure screen time for children during the pandemic and suggest that these associations may be particularly relevant for children with overweight/Obesity.
Mcguine et al [18] 2021, United States	Cross-sectional survey	N = 13002; M_age_ = 16.3years,SD = 1.2; Female:52.9%,Male:47%, other-prefer to not say:0.1%	May,2020	The participants were required to meet the following criteria: In grades 9 to 12, participate in club or interscholastic sports; High school across the United States were closed to in-person teaching,and many extracurricular activities were canceled.	The Pediatric Functional Activity Brief Scale score was highest (best) for grade 9 (mean = 14.5,[95% CI = 14.0,15.0])and lowest for grade 11 (mean = 10.9,[95% CI = 10.5,11.3]); The prevalence of depression symptoms was highest in team sport (74.1%) and lowest in individual sport (64.9%) participants;	The mental health, physical activity, and HRQoL(Health-related quality-of-life) of US adolescents during the COVID-19–related school closures and sport cancellations varied depending on sex, grade level, type of sport participation, and level of poverty.
Kang et al [26] 2021, China	Cross-sectional survey	N = 4898; M_age_ = 16.3years,SD = 1.3; Female:52%	March 8-March 15, 2020	All of the participants were from 49 middle schools in Yan’an, Shaanxi Province; During COVID-19 pandemic and school closures,all of the adolescents volunteered to participate in this study and completed an online questionnaire which was named International Physical Activity Questionnaire and Profile of Mood States.	Participants in this study accumulated 23.4±52.5 min of moderate-to-vigorous PA and 363.6±148.4 min of Sedentary time per day;The mood states of boys were better than that of girls (*p*<0.01);The mood states of students in Senior High School Grade 3 were at the highest level of mood disturbance (M = 105.8,SD = 11.2); High levels of PA were significantly associated with lower levels of total mood disturbance in this population(High PA group according to IPAQ: B = -3.22,SE = 0.40, *p*<0.001, Moderate: B = -1.47,SE = 0.37, *p*<0.001,compared to Low PA group).	Chinese adolescents maintained a sedentary lifestyle during the COVID-19 pandemic; Girls and students in Grade 3 Senior High School had higher level of mood disturbance; In Chinese adolescents, PA were significantly associated with mood states, with higher levels of PA during home isolation were related to more stable mood states.
Mcguine et al [16] 2022, United States	Cross-sectional survey	N = 559; Age_range_:13-19years, M_age_ = 15.7years,SD = 1.2; Female:43.6%	October,2020	All of the athletes from Wisconsin high school were recruited to participate in the study by completing an anonymous online survey; The participants were required to meet the following criteria: in grades 9 to 12 and plan to play interscholastic sports at their school, they also need to receive their parents’ permission.	The PLY group participants were less likely report moderate to severe symptoms of anxiety (PLY = 6.6%, DNP = 44.1%,p<0.001) and depression (PLY = 18.2%, DNP = 40.1%, *p*<0.001); They also demonstrated 41% higher (ie better) Pediatric Functional Activity Brief Scale scores (PLY = 23.2[95%CI = 22.0,24.5], DNP = 16.4 [95%CI = 15.5,17.8], *p*<0.001)and higher(better) Pediatric Quality of Life Inventory total scores (PLY = 88.4[95%CI = 85.9,90.9], DNP = 79.6 [95%CI = 76.8,82.4], *p*<0.001).	Adolescents athletes who able to return to sport participation in fall 2020 reported dramatically lower symptoms of anxiety and depression, higher physical activity levels,and Healthy-Related Quality of Life; This research suggest that sport participation during the COVID-19 pandemics is associated with significant mental and physical health benefits in adolescents.
Villodres et al [21] 2021, Spain	Cross-sectional survey	N = 899; Third year primary school education:n = 257,First year secondary school education:642; Female:53.8%	February,March and April 2021	Students were recruited from state and mixed funding schools in Spain; Participants were asked to fill out the same questionnaire twice in one day, the first time, they were asked to consider the period of total confinement or lockdown(15 March 2020–21 June 2020), the second time, they were asked to consider a time period prior to the onset of COVID-19.	Mean scores were obtained of 2.33±0.69 on the PAQ-C(use to evaluate physical activity), 29.67±6.28 on the Rosenberg scale(use to evaluate personal self-esteem); Both boys and girls reported less PA(2.33±0.69 vs. 2.87±0.72)and lower self-esteem(29.67±6.28 vs. 30.43±6.30) during total confinement compared to the pre-confinement period(*p*<0.001); A positive correlation was observed between PA and self-esteem (*r* = 0.091, *p*0 = 0.007), PA correlated negatively with BMI (*r* = −0.072; *p* = 0.031) and age (*r* = −0.191; *p*<0.001);	Students of pre-adolescent age were less likely to engage in healthy habits during the period of total confinement, negatively affecting their self-esteem; It was determined that girls engaged in less PA and had lower self-esteem than boys. This premise was found to already exist prior to confinement, although the strength of relationships increased during total confinement;
Xiao et al [19] 2021, China	Cross-sectional survey	N = 1680; Grades:7–12; Female:48.7%(n = 818),Male:51.3%(n = 862)	April,2020	The participants were from a large middle-high school located in Southwest China; they were volunteered to fill out a questionnaire during lockdown in early April 2020; The questionnaire was sent to students in grade 7 to 12 via a social media application named WeChat.	A higher grade was associated with a higher mood disturbance score, but not after adding the variable of at least 150 minutes of physical activity each week; Physical activity was significantly associated with a lower mood disturbance scores, but doing at least 150 minutes of physical activity each week significantly predicted a lower mood disturbance score by 10 points, which outweighed the effect of physical activity participating score; Under control other variables, participate at least 150 minutes of physical activity each week predicted fewer conflicts with parents.	Physical activity,particularly of at least 150 minutes’ duration each week, significantly decreased the likelihood of negative mood among adolescents during lockdown; Screen time,specifically other than that spent on online stud, had a negative association with mood, after controlling for the relevant variables(i.e., physical activity and body mass index); Less screen time and accumulating 150 minutes of physical activity were associated with fewer conflicts with parents.
McArthur et al [22] 2021, Canada	Cross-sectional survey	N = 846(mother-child dyad); Child: Age_range_:9-11years, M_age_ = 9.85years, SD = 0.78; Female: n = 398(47.1%)	May-August, 2020; During August 2008-December 2010, the mothers were recruited in pregnancy	All of the participants were recruited from Calgry, Canada; The state was in a state of emergency(COVID-19 pandemic), all of the school and children facilities were closures; 10% of families reported having a personal experience with COVID-19.	The score of anxiety(*r* = -0.08, *p*<0.01) and depression (*r* = -0.11, *p*<0.05)subscales of the BASC-2 at 8 years(maternal report) and physical activity were significant negative correlated; During COVID-19(July-August 2020), the score of anxiety subscales of the BASC-3(child self-report) and physical activity were significant negative correlated(*r* = -0.11, *p*<0.05), the score of happiness subscales of the BASC-3 (child self-report) and physical activity were significant positive correlated(*r* = 0.15, *p*<0.05); After controlling for child sex, age, and anxiety(Model 1), depression(Model 2), adaptive skills(Model 3) pre-COVID-19, physical activity was not a significant predictor of child anxiety, depression, happiness during COVID-19.	It appears that the more salient predictors of child mental health and well-being during COVID-19 were those more proximal, accessible, or directly experienced by the child (e.g., connectedness to caregivers, screen time, and sleep) versus ones that were more external to them (e.g., family income, maternal mental health, and peer interactions).
Tandon et al [29] 2021, United States	Cross-sectional survey	N = 1000; Age_range_:6-17years,M_age_ = 10.8, SD = 3.5; Female:46.7%; Age(range 6-10years): n = 500, M_age_ = 8.1,SD = 1.4, female:47.4%; Age(range 6-10years): n = 500, M_age_ = 14.0,SD = 2.0, female:49.4%	October 22-November2, 2020	During the COVID-19 pandemic; All data on children aged 6 to 10 years are based on parents reports; For children aged 11 to 17 years, parents were asked about family demographics and effects of COVID-19, children in this group were asked to self-report on their physical activity, screen time, and mental health.	COVID-19 stressors were significantly associated with higher total difficulties for both younger (β = 0.6; [95% CI = 0.3,0.9]) and older (β = 0.4; [95% CI = 0.0,0.7]) groups; After accounting for COVID-19 stressors, engaging in 7 d/wk (vs 0) of physical activity was associated with fewer externalizing symptoms in younger children (β = 2.0; [95% CI = −3.4,−0.6]);For older children, engaging in 1 to 6 and 7 day/week (vs 0) of physical activity was associated with lower total difficulties(β = −3.5 [95% CI = −5.3,−1.8])and(β = −3.6 [95% CI = −5.8,−1.4], respectively), fewer externalizing symptoms (β = −1.5 [95% CI = −2.5,−0.4] and(β = −1.3 [95%CI = −2.6,0], respectively), and fewer internalizing symptoms (β = −2.1[95%CI = −3.0, −1.1]) and β = −2.3[95%CI = −3.5, −1.1], respectively); More screen time was correlated with higher total difficulties among younger (β = 0.3; [95%CI = 0.1–0.5]) and older (β = 0.4;[95% CI = 0.2, 0.6])children.	More physical activity and less screen time were associated with better mental health for children, accounting for pandemic.
Mitra et al [23] 2021, Canada	Cross-sectional study	N = 932; Age_range:_9-15years	Spring 2020	All participants completed the survey during the COVID-19 outbreak; Parents filled out part of the survey where they provided information on socio-demographic characteristics and house hold changes during the pandemic (e.g., changes in income); Followed by that, one 9–15 year old child filled out the rest of the survey, where they self-reported their age, emotions, movement behaviours and other perceptions.	The majority of these children and youth self-reported a decline in healthy movements during the COVID-19 pandemic, with 56.3% reporting that they spent less time being physically active The top three self-reported changes are- feeling more bored (unpleasant and low activation), feeling calmer (pleasant and low activation), and feeling more worried (unpleasant and high activation); 49.4% children and youth were included in low pandemic-time SWB, 63.3% of them were likely to feel more bored during the pandemic, while 51.5% were likely to feel more worried. By contrast, only 1.7% were likely to feel more excited and 2.2% were likely to feel happier compared to the pre-pandemic period; 50.6% children and youth were included in high pandemic-time SWB, 56% of whom were likely to feel calmer and 52.1% felt happier during the pandemic. In contrast, only 0.7% would feel more alone or unsupported while 2.5% would feel sadder compared to the pre-pandemic period.	Having access to friends, indoor and outdoor spaces/ places to play and exercise, and healthy movement behaviors during the pandemic were correlated with improved SWB outcomes;
Ren et al [20] 2021, China	Cross-sectional study	N = 1487; Age_range:_10-17years, M_age_ = 13.14years SD = 1.55; Female:50%	April 19–26 2020	The survey was conducted during which the quarantine policy had ended but the schools remained closed. Students were recruited from four public schools in the urban area of Zhengzhou city, China; All adolescents were of Han ethnicity; The study was conducted with the consent of the participants and their guardian; Data were collected online; 11% of the adolescents lived in communities with confirmed or suspected COVID-19 cases during the quarantine, whereas 89% of the adolescents lived in communities without community infection.	Physical activity time (*β* = -0.07, *p*<0.001) and all four types of child routines (*β* ranging from -0.024 to -0.15, all *p*<0.001) were negatively associated with depressive symptoms; Adolescent age (*β* = 0.07, *p*<0.01), moderated the relation between community infection and adolescent depressive symptoms, to be specific, the associations between community infection and depressive symptom were significant for adolescents between the ages 12 and 17 years but were not significant for younger adolescents (ages 10 and 11years), physical activity time (*β* = -0.15, *p*<0.01), and daily living routines (*β* = -0.09, *p*<0.01) also moderated the relation between community infection and adolescent depressive symptoms; Adolescents who reported physical activity less than 0.5 hours(*β* = 0.41, *p*<0.001), 0.5–1 hour(*β* = 0.26, *p*<0.001) and 1–2 hours(*β* = 0.12, *p*<0.05)a day were more likely to have depression symptoms if COVID-19 was present in their community, but this was not the case among those who reported more than two hours physical activity a day or more;	Older adolescents are more psychologically susceptible to the presence of community infection than younger adolescents; Adolescent gender did not predict post quarantine depressive symptoms or moderate the relation between community infection and depressive symptoms; Daily physical activities and maintenance of daily living routines were two protective factors that buffered the association between community infection and adolescents’ depressive symptoms; Adolescents who have spent more time on physical activities during the quarantine may develop less post quarantine depressive symptoms associated with community infection; Leisure screen time use did not exacerbate the association between community infection and adolescent depressive symptoms.
Maximova et al [27] 2022, Canada	Cross-sectional study	N = 1095; Age_range:_9-12years; Female: n = 557(50.9%)	November-December 2020 & January-February 2021	All participants were from 20 schools located in British Columbia, Alberta, Manitoba, and Northwest Territories to participate in the survey; Survey were conducted in school during regular class time; Participants were asked to report their physical activity level during the Spring 2020 lockdown and pre-COVID-19.	About two-thirds of girls and boys (62% and 64%, respectively) recalled their physical activity levels to be lower during the lockdown than before the lockdown; 44% and 31% of girls and boys perceived, on average, their mental health and wellbeing to be worse, the majority(56% and 69%) perceived their mental health and wellbeing to be better during the lockdown; 32% of students reported feeling lonely the same as before the lockdown; Students who cared about being physically active were more likely to report no increases in time playing video games and using a cellphone and were more likely to maintain positive mental health;	Girls who were more physically active during than before the lockdown were less likely to experience ‘internalizing and functioning problems, tiredness and loneliness’ and more likely to have a ‘positive outlook on future and time during lockdown’ relative to those who were less physically active; In girls, spending less or the same amount of time playing video games was associated with a higher likelihood of maintaining positive mental health and wellbeing during the lockdown and being bored and lonely; Boys who were physically active during the lockdown were more likely to have a‘positive outlook on future and time during the lockdown’.
Alves et al [30] 2020, American	Cross-sectional study	N = 65; Age_range_:9-15years; Female: n = 40(61.5%)	April 20–26 2020 & June 26 2020	Of all the participants, 38 (58.4%) children had gestational diabetes exposure; The survey was completed during the outbreak lockdown; Participants were recruited from the existing observational Brain Child study on neuroendocrine programming associated with GDM-exposure; Children were born at a Kaiser Permanente Southern California (KPSC) and had no history of significant medical disorders.	The proportion of children who engaged in any VPA was significantly lower for GDM-exposed children (5%) compared to unexposed children (30%) with an odds ratio (OR) of 7.6(95% Wald CL:1.5–39.3 *p* = 0.02); Children who engaged in VPA (N = 10), had lower mean S-anxiety compared to children who did not engage in VPA (N = 54), (*β* = -2.8, 95% CL:−5.1 to −0.6, *p* = 0.01); Children who reported more time spent in MVPA also had lower S-anxiety (*β* = -0.2, 95% CL: −0.4 to −0.1, *p* = 0.01); In the post-hoc mediation analysis, accounting for VPA levels explained 75% (Wald 95% CL: −0.8 to 150.9, P = 0.05) of the relationship between GDM-exposure and S-Anxiety.	During the pandemic, children exposed to GDM reported higher anxiety symptoms and less engagement in VPA compared to unexposed Children; The relationship between GDM exposure and greater anxiety in children was partially explained by lower engagement in VPA; engaging in VPA During stressful periods is associated with reduced anxiety levels, and that children exposed to GDM could especially benefit from engaging in VPA; The potential adverse effects of GDM-exposure on child mental health during the pandemic exhibit relationships with physical activity levels.

The 14 studies, through quantitative methods, evaluate the level of the correlation between PA and mental health of children and adolescents. They evaluate the intensity, duration, and frequency of PA. In the quantitative analysis, they adopt International PA Questionnaire (IPAQ) [26], the Pediatric Functional Activity Brief Scale (PFABS) [16, 18], the Leisure-Time Exercise Questions [25], questions from the Youth Risk Behavior Surveillance Survey [29], and the PA questionnaire for children (PAQ-C) [21, 27]. As for mental health, most of the studies evaluate people’s anxiety, depression, and other negative emotions (such as tension, depression, anger, fatigue, confusion, etc.) through the State-Trait Anxiety Inventory for state-Anxiety for Children(STAIC S-Anxiety) [28], the 10-item Positive and Negative Affect Schedule for Children (PANAS-C)^1^, Profile of Mood States (POMS) [19, 26], the General Anxiety Disorder 7-Item(GAD-7) for anxiety [16, 18], Patient Health Questionnaire 9-Item (PHQ-9) for depression [16, 18], the Strengths and Difficulties Questionnaire (SDQ) [29], World Health Survey [24], Behavior Assessment System for Children (BASC-3) [22], Middle Years Development Instrument (MDI) [22], Kessler‑10 (K10) [25], the Rosenberg self-esteem scale [21], and the 20-item Center for Epidemiologic Studies Depression [20]. Besides, Xiao et al [19] also study the correlation between PA and conflicts with parents, which can reflect the influence on the mental health of children and adolescents to a certain extent. Though these papers use different measurements, they are recognized for their validity.

## Mental health

### Negative affect

7 studies, come from America, China, Spain, Canada, and Pakistan, focus on the negative influence on the mental health of children and adolescents during the COVID-19 pandemic, which show that many people have some kinds of mental symptoms [19–22, 25, 26, 28] such as anxiety, depression, boredom, emotional disorders, decreased self-esteem, etc. that are even more severe than before the lockdown. The symptoms that are most frequently seen are anxiety, depression, and emotional disorders.

The increase in anxiety is presented in 4 studies from China, America, and Canada [19, 22, 23, 28]. Among them, 2 studies from America show that girls are more likely to have medium and severe anxiety and students in the senior year are more likely to have medium and severe anxiety than students of other grades. 3 from Pakistan, Canada, and America show increased depression of children and adolescents during the COVID-19 pandemic [20, 22, 25]. An increase in emotional disorders is shown in 3 from Brazil and China [19, 24, 26], especially for girls [19, 24, 26, 27]. In China, students in the senior year have more severe emotional disorders than any students from other grades [19, 26]. There is a positive correlation between age and emotions like sadness, anger, loneliness, etc. in students from Brazil [24]. While 1 study from Spain shows that boys have higher self-esteem than girls during the COVID-19 pandemic [21]. In addition, the pandemic makes some children and adolescents have more external emotions like anger. 1 study from America finds that after the outbreak of the COVID-19 pandemic, the group with older age (11–17) is more likely to have internal emotions like anxiety, depression, loneliness, etc. than the group with younger age (6–10) [29].

### Positive affect

A research from Canada finds that some children and adolescents become happier and more excited during the pandemic. There are 1095 students (came from grade 4–6, aged 9–12), who come from 20 schools located in communities with poor economic conditions in northern Canada, having improvements in mental health and general happiness during the lockdown. While 32% of students think they feel the same loneliness before and during the lockdown.

### Physical activity

Four studies from Spain, Canada, America, and China pay attention to the decrease in PA among children and adolescents caused by the pandemic [16, 23, 26, 27]. Among them, children and adolescents in the United States and Canada reduced their physical activity and increased their screen time [23, 28, 29], while children and adolescents in China spent less time in physical activity and more time sitting during the COVID-19 [26], and physical activity is negative correlation with these two factors [26]. In terms of gender, boys spend more time on PA than girls, especially on moderate and vigorous activity [18, 19, 21, 26]. Chinese girls spend less time on PA [32]. But there are studies show that there is no significant difference in gender relating to PA during the pandemic (*P* = 0.238) [21]. It is recognized that both boys and girls Boys and girls are spending less time physically active than before and spend more time in front of screens, which can be found in 2 studies from China and Spain [19, 21]. In terms of age, 2 from China and Spain find that the younger the adolescents from China and Spain are, the more physical activities they have [19, 21]. In addition, the PFABS score for American group players having PA during the pandemic is lower than that of American individual players [18].

### Accosiation between mental heath and physical activity during COVID-19

Of the 14 studies studying the connection between PA and the mental health of children and adolescents during the pandemic, which come from America, China, Spain, and Pakistan, Ten studies [16, 18, 19, 21, 24–26, 28–30] all support that PA under COVID-19 is beneficial to the mental health of children and adolescents to some extent, especially in improving anxiety, depression and other negative emotions.

### Anxiety

Four studies from America find that PA helps reduce anxiety during the COVID-19 pandemic. Among overweight or obese children aged 9–12, a longer time of MVPA is connected with less anxiety (revised the age, gender, social economic status of the children and the exposure condition of mother GDM, Pearson R = -0.52, *P*<0.05) [28]. Children and adolescents aged 9–15 with MVPA, or with more time on MVPA, have a lower score on anxiety compared with those with no MVPA, or with less time on MVPA (adjusted β1 = -2.8, P = 0.01; β1 = -0.2, P = 0.01). A study [29] shows that having PA more than 1 day each week helps improve internal emotions like depression and anxiety for adolescents aged 11–17. Another study sets two groups. One is the intervention group, which consists of adolescent players aged 13–19 who participate in exercise during the pandemic. The other is the control group, which consists of those who do not exercise. The study finds that the intervention group shows fewer symptoms of moderate or severe anxiety [16].

### Depression

Three studies from Pakistan, China, and America find that PA helps reduce depression during the COVID-19 lockdown. The research from Pakistan [25] suggests that there is a negative correlation (*R* = 0.34, *P*<0.01) between the score of PA and depression among adolescents aged 15–19. A study [20] shows that when adolescents aged 10–17 spend more time on PA, they will have less depression caused by the pandemic. Another study [29] indicates that having PA over one day each week helps to improve internal symptoms like depression and anxiety among adolescents aged 11–17. A study from America takes adolescent players aged 13–19 as the object of observation, and sets the intervention group, consisting of individual players (who are easier to have PA during the pandemic), and the control group, consisting of group players. It finds that the intervention group is not easy to have symptoms of depression during the pandemic (64.9% VS 74.1%) [18].

### Mood and emotion

Three studies from China and Brazil find a connection between PA and emotions. A research [26] suggests that a higher level of PA is connected with a lower intervention of general emotions (MVPA: adjusted B = -1.47, *P*<0.001; HVPA: B = -3.22, *P*<0.001). Another research [19] shows that having PA for at least 150 minutes each week can significantly reduce the possibility of adolescents aged 7–12 having negative emotions during the pandemic (R = -10.98, *P*<0.001). And a research [24] finds that for adolescents aged 12–17, having PA twice a week, each time over 1 hour, is negatively correlated with having negative emotions like sadness, irritability, etc. (adjusted OR = 0.88, *P* = 0.004).

### Other mental symptoms

In addition, three papers from Spain, America, and Canada explore self-esteem, external manifestation, and subjective happiness. The latest research from Spain [21] shows that there is a significant correlation between the self-esteem of adolescents aged 10–14 and PA during the pandemic(R = 0.091; *P* = 0.007). A study [29] shows that for children aged 6–10, having PA seven days a week can improve their external behavior like impulsion, violence, etc. (βcoefficient, −2.0; 95% CI, −3.4 to −0.6). While for adolescents aged 11–17, having PA over one day a week can improve their external behavior like impulsion, violence, etc. A study from Canada explores PA and the place at the same time. It finds that for adolescents aged 9–15, having PA regularly or having enough place to exercise indoors or outdoors is connected with subjective happiness [23].

### Duration of physical activity

Four studies from China, America, and Brazil show that the influence of PA on the mental health of children and adolescents may depend on enough time for exercising. A research [19] shows that having PA for at least 150 minutes each week can significantly reduce the possibility of children and adolescents aged 7–12 having negative emotions during the lockdown. Another research [29] suggests that for children aged 6–10, having PA seven days a week can improve their external behavior like impulsion, violence, etc. While for adolescents aged 11–17, having PA over one day a week can improve their external behavior like impulsion, violence, etc., and internal symptoms like depression, anxiety, etc. And a research [24] indicates that for adolescents aged 12–17, having PA twice a week, each time over 1 hour, is negatively correlated with negative emotions like sadness, irritability, etc. Another research [20] finds that for adolescents aged 10–17, spending more time on PA helps reduce depression caused by the pandemic.

### Negative outcome

Five studies from America, China, and Canada [19, 22, 27–29] suggest that PA may not improve some mental symptoms for children during the pandemic, and these irrelevant results are found in children under 12. The research [28] shows that for children with a healthy weight, there may not be a correlation between MVPA and the score of anxiety, positive emotions, and negative emotions of children aged 9–12 during the pandemic (34 cases, revised *P*>0.05). In addition, though for children who are overweight or obese, a longer time of MVPA is connected with less anxiety, PA cannot increase their positive emotions. The research [19] finds that the pure score of PA is not connected with the negative emotions of children aged 7–12 during the pandemic. Unless the time of PA exceeds 150 minutes each week, PA cannot reduce negative emotions. A research from America [29] has the same finding. For children aged 6–10, having PA 1–6 days a week cannot improve their mental health. Only when they exercise 7 days a week can their external behavior like impulsion, violence, etc., be improved, which, however, does not help improve internal symptoms like depression, anxiety, etc. The research [22] finds that after revising the age and symptoms of mental health prior to the pandemic, the correlation between PA and anxiety, depression, and the sense of happiness of adolescents aged 9–11 during the pandemic is not statistically significant (*P*>0.05). What is connected to mental health is the contact with the caregiver, sleep quality, and the time spent in front of screens. The research [27] shows that for children aged 9–12, the correlation between PA before the pandemic and loneliness and boredom is statistically significant.

## Discussion

We present a detailed systematic review of the association of PA on the Mental Health of Children and Adolescents during s COVID-19 pandemic-induced school closures. All included studies were the effects of physical activity on mental health during school closures enacted lockdown in the COVID-19. Investigating the association between PA and mental health in this specific demographic group. The majority of these studies (n = 10, 71.4%) consistently indicate that participation in PA positively affects mental health [16, 18, 19, 21, 24–26, 28–30]. Moreover, compelling evidence substantiates that maintaining a regular exercise routine during this crisis can improve psychological well-being by alleviating negative emotions like anxiety and depression. There is a strong theoretical case to be made that school closures caused by the pandemic may have caused a range of harms, particularly mental health hazards, by reducing social contact with peers and teachers [9, 34], It is recognized that childhood and adolescence are key periods of physical and mental development, as well as a period of physical and mental fragility and vulnerability. The theory of positive youth development, based on the social ecosystem theory, proposes that PA provide children and adolescents with opportunities to develop relationships, that participation in it can enhance their confidence, competence, character, caring and connection, and that they have a lower risk of academic, psychological, social and behavioral problems [31–33]. In fact, early childhood environments play an important role in promoting PA development because these environments often have the resources to implement PA [34].

Studies findings have demonstrated that overweight or obese children can alleviate anxiety by engaging in MVPA for extended durations (*P*<0.05) [28]. For individuals aged 9–15, participation in VPA (Vigorous Physical Activity) or increasing overall levels of PA is associated with lower anxiety scores (*P*<0.01) and increased happiness. Additionally, adhering to regular PA routines and having sufficient indoor/outdoor space for exercise may mitigate the development of depression and anxiety symptoms caused by epidemic stress [18, 20, 23]. Evidence suggests a negative correlation between the duration and intensity of PA among adolescents aged 15–17 and rates of depression (*P*<0.01). Engaging in sports at least twice a week can enhance external behaviors such as impulsive violence while reducing internal psychological symptoms like depression and anxiety. Participating in more than one hour of PA on two separate occasions each week can effectively decrease negative emotions such as sadness and irritability (*P*<0.05) [24, 25, 29]. Furthermore, during the epidemic period, young athletes aged 13–19 who participated in individual sports exhibited lower likelihoods of experiencing moderate to severe anxiety symptoms compared to those engaged in team sports; furthermore, individual sports were found to be more effective at alleviating depression [16, 18]. High levels of PA were also linked to reduced total emotional disturbance levels (*P*<0.001), while engaging in at least 150 minutes of weekly PA significantly decreased the probability of negative emotions among children aged 7–12 during lockdowns [19].The frequency of engaging in exercise for at least two days per week was also found to be associated with enhanced external behaviors, such as reduced impulsive violence, among individuals aged 15–17 [24, 25, 29]. Furthermore, the epidemic has posed limitations on the availability of suitable spaces and low socioeconomic status has hindered the implementation of sports activities [30].

The mood effect theory of exercise points out that physical exercise can improve and treat individual anxiety and depression [35, 36]. The reason may be that exercise can increase the release of serotonin; Growth neuron expression factors such as brain-derived neurotrophic factor (BDNF), which play a very important role in the development and maintenance of the peripheral and central nervous system [37], can supply a variety of neurons that regulate emotional behavior, such as cholinergic, dopaminergic and 5-hydroxytryptaminergic neurons. Or the central nervous system, such as the hippocampus, neocortex, amygdala, cerebellum and hypothalamus distributed in the central nervous system, which are key brain areas regulating emotional behavior, nutritional support [29]. In addition, physical exercise can also promote the production and release of beta-endorphins in the human body, reduce activities such as adrenaline and cortisol [38], stimulate cognitive thinking and emotional cognition, and thus reduce negative emotions such as depression, anxiety and stress in adolescents and children.

Meanwhile, compared with males, females have a higher level of anxiety and depression and a lower level of body movement and HRQoL(health-related quality of life) score. Though there is a difference in gender, the level of depression and anxiety of both males and females is higher than before the pandemic. On the other hand, compared with individual athletes, team athletes showed more severe symptoms of anxiety and depression and lower levels of PA and HRQoL during lockdown, which may be due to the ability of individual athletes to continue to participate in PA or exercise even when space is restricted [39].

However, not all included cross-sectional surveys supported the positive effects of PA on mental health. For example, one study revealed that MVPA may not be associated with positive or negative emotions in healthy weight children aged 9–12 years during the pandemic (*P*>0.05) [28]. PA higher than the pre-epidemic level did not indicate a significant correlation with loneliness, boredom and other negative emotions (*P*>0.05) [27]. Even after adjusting for age, pre-epidemic mental health and other symptoms, PA was not correlated with anxiety, depression and happiness in 9–11 year olds during the epidemic period (*P*>0.05). On the contrary, caregiver contact, children’s sleep status and screen time were related to their mental health level (*P*<0.05) [22].

There is a "conditional" support for the impact of PA on the mental health of children and adolescents in the context of COVID-19. It takes a certain amount of time or intensity of PA to have a beneficial effect. However, other studies have shown that any PA during the COVID-19 pandemic can help improve an individual’s mental health, and its effect does not depend on factors such as the frequency and intensity of exercise. In other words, as long as individuals engage in PA during isolation, it will promote their mental health [40].

One study suggested that the weekly exercise duration should be at least 150 minutes to reduce the interference of negative emotions, and the simple PA score had no correlation with the negative emotions of 7–12 years old during the epidemic period (*P*>0.05) [19].Similarly, although it was found that children aged 6–10 years old needed to exercise 7 days a week to improve their external behaviors such as impulsive violence, it still could not improve their internal psychological symptoms such as depression and anxiety [29].

A possible explanation for these different findings is that there is no quality rating for PA or exercise. In 11 valid cross-sectional surveys, study duration ranged from 1 week to 4 months, with more than half of the studies lasting longer than 2 months. Given the limited number of studies included in the review, more cross-sectional investigations are needed to strengthen the evidence base and confirm PA dose (i.e. duration, intensity, frequency, etc.). Overall, the evidence that PA promotes mental health during the pandemic is strong.

Mental health problems are a leading cause of health-related disability in children and adolescents [41]. Worldwide, approximately 10–20% of children and adolescents experience mental health problems [42], and mental health problems in childhood or adolescence can also have an impact in adulthood, such as mood disorders or employment difficulties [43]. Schools were closed during the pandemic, and most family activities and group extracurricular activities were canceled. School routines are essential coping mechanisms for young people with mental health problems. During school closures, children lose a pillar of their lives, and their symptoms may recur. For children and adolescents with mental health issues, suspension also means that they do not have access to resources through school [44]. The current global pandemic has led to a significant increase in the amount of time children and adolescents spend online and on social media [45]. Studies have shown that the excessive use of smart phones/Internet can lead to mental or behavioral problems. In addition to poor academic performance, the reduction of social interaction in real life and the neglect of personal life can also lead to interpersonal relationship disorders and emotional disorders [46, 47].Meanwhile, the accompanying sedentary behavior is more likely to cause anger, fatigue, depression and other negative emotions in children and adolescents [48]. The findings also showed that mental health, PA, and HRQoL scores also deteriorated with grade level, which may be related to higher grade students’ greater perception of the end of their careers [39]. Therefore, we explored possible preventive factors for reducing mental health problems and promoting good mental health, revealing that participation in PA may have some positive effects on the mental health of children and adolescents, which may depend on the breadth, intensity and duration of participation in PA.

Our data do not allow us to examine whether providing online PA mitigated some of the associations of during COVID-19 pandemic-induced school closures. Online PA does provide a degree of social interaction with exercise intensity and may act to mitigate some of the harms caused by reducing social mixing. It is suggested that further research is needed on its relationship with mental health in the future.

### Limitations

Strengths of the study were the use of a large number of educational as well as health electronic databases and preprints and independent assessment of study eligibility, data extraction, and quality. This review also has several limitations. First, we were unable to study the influence of school closure distinct from broader social lockdown, and thus our findings relate to lock downs including school closures. Second, none of the results included in this review mention specific exercise methods or types of exercise (e.g. Calories or what kind of exercise/physical activity) are effective in improving the mental health of children and adolescents in the face of COVID-19. But creating a control group without intervention is morally questionable, which can help prove which type of activity can effectively improve the mental health of children and adolescents. Third, the studies were cross-sectional, providing weak evidence. Many publications included only simple analyses that did not take account of potential confounders. Fourth, most of the studies are based on online questionnaires and telephone interviews, the cross-sectional study helps to understand the direct influence or short-term effects at a specific time. However, the limitation of the cross-sectional study is that these studies cannot generate a conclusion about the long-term influence of the pandemic and many publications included only simple analyses that did not take account of potential confounders. Fifth, because of heterogeneity of study designs and measures, it was not possible to conduct a meta-analysis; rather, the results were summarized with a narrative synthesis. Sixth, we extensively searched multiple databases using relevant software management systems, but may have missed the studies. In the end, considering the small number of empirical studies, the conclusive statement that PA can improve the mental health of children and adolescents should be based on more research basis in the future.

## Conclusions

In this systematic review of reports from the COVID-19 pandemic-induced school closures, reports that PA has a improve on the mental health of children and adolescents to a certain extent. it is found that PA may be helpful in reducing mental health symptoms of children and adolescents who are influenced by class suspension because of the COVID-19 pandemic. Therefore, stakeholders of the mental health of children and adolescents around the world should recommend PA because it is a practicable and beneficial way for long-term mental support.

## Supporting information

S1 Checklist PRISMA 2020 checklist. (PDF)

S1 File (DOC)
