# Supplementary material for: Physical activity on the mental health of children and adolescents during COVID-19 pandemic-induced school closures—A systematic review
Source: PLoS One. 2024 Jun 25;19(6):e0299158. doi: 10.1371/journal.pone.0299158 (PMC11198782; doi:10.1371/journal.pone.0299158)
Supplement: S1 File — (DOC) [file pone.0299158.s002.doc]

PubMed: 409

(((child, preschool[mh]) OR (adolescent[mh]) OR (child[tiab] OR children[tiab] OR child’s[tiab] OR "young people"[tiab] OR "young person*"[tiab] OR youth*[tiab] OR infant*[tiab] OR schoolchild*[tiab] OR "school child*"[tiab] OR boy*[tiab] OR girl*[tiab] OR teen*[tiab] OR adolescen*[tiab] OR pediatric*[tiab] OR paediatric*[tiab] OR peadiatric*[tiab])) AND (physical activity OR physical fitness OR physical education OR exercise* OR motor activity OR walk* OR jog* OR sedentary* OR sitting) AND ("physiological outcomes" OR "psychological outcomes" health OR "health behavior" OR lifestyle OR health-related OR "quality of life" OR health status OR mental health )) AND (("education access"[tiab] OR "access to education"[tiab]) OR (("primary education"[tiab] OR "secondary education"[tiab]) AND ("no access"[tiab] OR "restricted access"[tiab] OR restrict*[tiab])) OR (quarantine*[tiab] OR self-isolat*[tiab] OR "lock down*"[tiab] OR lockdown*[tiab] OR lock-down*[tiab] OR "class dismissal*"[tiab] OR "school dismissal*"[tiab] OR "social distanc*"[tiab] OR "stay at home" [tiab] OR "shut down*"[tiab] OR shut-down*[tiab] OR shutdown*[tiab] OR "staying at home"[tiab]) OR (((schools[mh:noexp]) OR (schools[mh]) OR (school*[tiab] OR preschool*[tiab] OR "pre school*"[tiab] OR preschool*[tiab] OR kindergarten*[tiab] OR kindergarden*[tiab]))) AND ((close[tiab] OR closed[tiab] OR closure[tiab] OR closures[tiab] OR closing[tiab])))

Scopus: 200

( ( TITLE-ABS-KEY ( ( "physiological outcomes" OR "psychological outcomes health" OR "health behavior" OR lifestyle OR health-related OR "quality of life" OR health AND status OR mental AND health ) ) ) AND ( TITLE-ABS-KEY ( ( physical AND activity OR physical AND fitness OR physical AND education OR exercise OR motor AND activity OR walk OR jog OR sedentary OR sitting ) ) ) AND ( TITLE-ABS-KEY ( ( ( child, AND preschool ) OR ( adolescent ) OR ( child OR children OR child’s OR "young people" OR "young person" OR youth OR infant OR schoolchild OR "school child" OR boy OR girl OR teen OR adolescen OR pediatric OR paediatric OR peadiatric ) ) ) ) ) AND ( TITLE-ABS-KEY ( ( ( ( schools ) OR ( schools ) OR ( school* OR preschool* OR "pre school*" OR preschool* OR kindergarten* OR kindergarden* ) ) ) AND ( ( close OR closed OR closure OR closures OR closing ) ) OR ( quarantine* OR self-isolat* OR "lock down*" OR lockdown* OR lock-down* OR "class dismissal*" OR "school dismissal*" OR "social distanc*" OR "stay at home" OR "shut down*" OR shut-down* OR shutdown* OR "staying at home" ) OR ( "education access" OR "access to education" ) OR ( ( "primary education" OR "secondary education" ) AND ( "no access" OR "restricted access" OR restrict* ) ) ) )

CINAHL: 460

S1 TI ((child, preschool) OR (adolescent) OR (child OR children OR child's OR "young people" OR "young person" OR youth OR infant OR schoolchild OR "school child" OR boy OR girl OR teen OR adolescen OR pediatric OR paediatric OR peadiatric)) 559900

S2 TI (physical activity OR physical fitness OR physical education OR exercise OR motor activity OR walk OR jog OR sedentary OR sitting) 106731

S3 TI ("physiological outcomes" OR "psychological outcomes" health OR "health behavior" OR lifestvle OR health-related OR "qualitv of life" OR health status OR mental health) 135577

S4 S1 AND S2 AND S3 460

Cochrane: 1238

#1((child, preschool) OR (adolescent) OR (child OR children OR child’s OR "young people" OR "young person" OR youth OR infant OR schoolchild OR "school child" OR boy OR girl OR teen OR adolescen OR pediatric OR paediatric OR peadiatric)) 352,249

#2 (physical activity OR physical fitness OR physical education OR exercise OR motor activity OR walk OR jog OR sedentary OR sitting) 216,055

#3(“physiological outcomes” OR “psychological outcomes health” OR “health behavior” OR lifestyle OR health-related OR “quality of life” OR health status OR mental health) 260,936

#4(“education access” OR “access to education”) OR ((“primary education” OR “secondary education”) AND (“no access” OR “restricted access” OR restrict*)) 128

#5(quarantine* OR self-isolat* OR "lock down*" OR lockdown* OR lock-down* OR "class dismissal*" OR "school dismissal*" OR "social distanc*" OR "stay at home" OR "shut down*" OR shut-down* OR shutdown* OR "staying at home") 1061

#6(((schools) OR (schools) OR (school* OR preschool* OR "pre school*" OR preschool* OR kindergarten* OR kindergarden*))) AND ((close OR closed OR closure OR closures OR closing)) 6409

#7 #4 OR #5 OR #6 7481

#8 #1 AND #2 AND #3 AND #7 1238

Cochrane Library

| 1 | ((child, preschool) OR (adolescent) OR (child OR children OR child’s OR "young people" OR "young person" OR youth OR infant OR schoolchild OR "school child" OR boy OR girl OR teen OR adolescen OR pediatric OR paediatric OR peadiatric)) | 352,249 |
| --- | --- | --- |
| 2 | (physical activity OR physical fitness OR physical education OR exercise OR motor activity OR walk OR jog OR sedentary OR sitting) | 216,055 |
| 3 | (“physiological outcomes” OR “psychological outcomes health” OR “health behavior” OR lifestyle OR health-related OR “quality of life” OR health status OR mental health) | 260,936 |
| 4 | (“education access” OR “access to education”) OR ((“primary education” OR “secondary education”) AND (“no access” OR “restricted access” OR restrict*)) | 128 |
| 5 | (quarantine* OR self-isolat* OR "lock down*" OR lockdown* OR lock-down* OR "class dismissal*" OR "school dismissal*" OR "social distanc*" OR "stay at home" OR "shut down*" OR shut-down* OR shutdown* OR "staying at home") | 1061 |
| 6 | (((schools) OR (schools) OR (school* OR preschool* OR "pre school*" OR preschool* OR kindergarten* OR kindergarden*))) AND ((close OR closed OR closure OR closures OR closing)) | 6409 |
| 7 | #4 OR #5 OR #6 | 7481 |
| 8 | #1 AND #2 AND #3 AND #7 | 1238 |

SPORTDiscus: 291

S1 TI (child, preschool) OR (adolescent) OR (child OR children OR child's OR "young people" OR "young person" OR youth OR infant OR schoolchild OR "school child" OR boy OR girl OR teen OR adolescen OR pediatric OR paediatric OR peadiatric)) 84.273

S2 TI (physical activity OR physical fitness OR physical education OR exercise OR motor activity

OR walk OR jog OR sedentary OR sitting) 135,074

S3 TI ("physiological outcomes" OR "psychological outcomes health" OR "health behavior" OR lifestyle OR health-related OR "quality of life" OR health status OR mental health ) 13,429

S4 S1 AND S2 AND S3 291

Embase: 405

#1 'physical activity' :ab,ti OR 'physical fitness':ab,ti OR 'physical education':ab,ti OR exercise:ab,ti OR 'motor activity': ab, ti OR walk:ab,ti OR jog:ab,ti OR sedentary:ab,ti OR sitting: ab,ti 711,592

#2 'child, preschool' ab,ti OR adolescent:ab,ti OR children:ab,ti OR child:ab,ti OR 'young people :.ab,ti OR 'young person':ab,ti OR youth:ab,ti OR infant:ab,ti OR schoolchild:ab,ti OR 'school child":ab,ti OR boy: ab,ti OR girl:ab,ti OR teen:ab,ti OR adolescen:ab,ti OR pediatric: ab,ti OR paediatric:ab,ti OR peadiatric:ab,ti 2,737,565

#3 'physiological outcomes' :ab,ti OR 'psychological outcomes health':ab,ti OR 'health behavior' :ab,ti OR lifestyle:ab,ti OR 'health related' : ab,ti OR "quality of life' :ab,ti OR 'health status' :ab,ti OR 'mental health'.ab.ti 1,077,847

#4 'education access' :ab,ti OR 'access to education':ab,ti OR (('primary education':ab,ti OR 'secondary education':ab,ti) AND ('no access :ab,ti OR 'restricted access' :ab,ti OR restrict* :ab, ti))

#5quarantine*:ab,ti OR 'self isolat**:ab,ti OR lockdown*:ab ti OR 'lock down* :ab,ti OR 'class dismissal** :ab,ti OR 'school dismissal* :ab,ti OR 'social distanc* :ab,ti OR 'stay at home' :ab,ti OR 'shut down* ab,ti OR shutdown*:ab,ti OR 'staying at home': ab,ti 49,829

#6 (schools:ab,ti OR school*:ab,ti OR 'pre school* :ab,ti OR preschool*: ab,ti OR kindergarten*:ab,ti OR kindergarden*:ab,ti) AND (close:ab,ti OR closed:ab,ti OR closure:ab,ti OR closures: ab,ti OR closing: ab,ti) 12,501

#7 #4 OR #5 OR #6 62,446

#8 #1 AND #2 AND #3 AND #7 405

psyclNFO: 109

Abstract: ("physiological outcomes" OR "psychological outcomes health" OR "health behavior" OR lifestyle OR health-related OR "quality of life" OR health status OR mental health) AND ((abstract: (physical activity) OR abstract:(physical fitness) OR abstract: (physical education) OR abstract: (exercise) OR abstract: (motor activity) OR abstract: (walk) OR abstract: (jog) OR abstract: (sedentary) OR abstract: (sitting))) AND (((abstract: (child, preschool)) OR (abstract:(adolescent)) OR (abstract: (child) OR abstract:(children) OR abstract: (child's) OR abstract:("young people") OR abstract: ("young person") OR abstract: (youth) OR abstract: (infant) OR abstract:(schoolchild) OR abstract: ("school child") OR abstract: (boy) OR abstract: (girl) OR abstract:(teen) OR abstract: (adolescen) OR abstract:(pediatric) OR abstract: (paediatric) OR abstract:(peadiatric))))

WOS: 882

#1 TS=(((child, preschool) OR (adolescent) OR (child OR children OR child’s OR "young people" OR "young person" OR youth OR infant OR schoolchild OR "school child" OR boy OR girl OR teen OR adolescen OR pediatric OR paediatric OR peadiatric)) ) 2,998,669

#2 TS=((physical activity OR physical fitness OR physical education OR exercise OR motor activity OR walk OR jog OR sedentary OR sitting) ) 1,320,358

#3 TS=((“physiological outcomes” OR “psychological outcomes health” OR “health behavior” OR lifestyle OR health-related OR “quality of life” OR health status OR mental health)) 1,293,778

#4 TS=( (“education access” OR “access to education”) OR ((“primary education” OR “secondary education”) AND (“no access” OR “restricted access” OR restrict*)) ) 3,059

#5 TS=((quarantine* OR self-isolat* OR "lock down*" OR lockdown* OR lock-down* OR "class dismissal*" OR "school dismissal*" OR "social distanc*" OR "stay at home" OR "shut down*" OR shut-down* OR shutdown* OR "staying at home")) 90,634

#6 TS=((((schools) OR (schools) OR (school* OR preschool* OR "pre school*" OR preschool* OR kindergarten* OR kindergarden*))) AND ((close OR closed OR closure OR closures OR closing)) ) 22,984

#7 #4 OR #5 OR #6 114,809

#8 #1 AND #2 AND #3 AND #7 882
